# Epigallocatechin Gallate Modulates Essential Elements, Zn/Cu Ratio, Hazardous Metal, Lipid Peroxidation, and Antioxidant Activity in the Brain Cortex during Cerebral Ischemia

**DOI:** 10.3390/antiox11020396

**Published:** 2022-02-16

**Authors:** Ming-Cheng Lin, Chien-Chi Liu, Yu-Chen Lin, Ching-Wen Hsu

**Affiliations:** 1 Department of Medical Laboratory Science and Biotechnology, Central Taiwan University of Science and Technology, Taichung 406053, Taiwan; 2 Department of Nursing, National Taichung University of Science and Technology, Taichung 404336, Taiwan; vickyliu@gm.nutc.edu.tw; 3 Department of Medicine, Chung Shan Medical University, Taichung 402306, Taiwan; s0601139@gm.csmu.edu.tw; 4 Department of Pharmacy, Chung Kang Branch, Cheng Ching Hospital, Taichung 407211, Taiwan

**Keywords:** EGCG, cerebral ischemia, antioxidant, essential element, hazardous metal

## Abstract

Cerebral ischemia induces oxidative brain injury via increased oxidative stress. Epigallocatechin gallate (EGCG) exerts anti-oxidant, anti-inflammatory, and metal chelation effects through its active polyphenol constituent. This study investigates whether EGCG protection against cerebral ischemia-induced brain cortex injury occurs through modulating lipid peroxidation, antioxidant activity, the essential elements of selenium (Se), zinc (Zn), magnesium (Mg), copper (Cu), iron (Fe), and copper (Cu), Zn/Cu ratio, and the hazardous metal lead (Pb). Experimentally, assessment of the ligation group was performed by occlusion of the right common carotid artery and the right middle cerebral artery for 1 h. The prevention group was intraperitoneally injected with EGCG (50 mg/kg) once daily for 10 days before cerebral ischemia. The brain cortex tissues were homogenized and the supernatants were harvested for biochemical analysis. Results indicated that cerebral ischemia markedly decreased SOD, CAT, Mg, Zn, Se, and Zn/Cu ratio and increased malondialdehyde (MDA), Fe, Cu, and Pb in the ischemic brain cortex. Notably, pretreating rats with EGCG before ischemic injury significantly reversed these biochemical results. Our findings suggest that the neuroprotection of EGCG in the ischemic brain cortex during cerebral ischemia involves attenuating oxidative injury. Notably, this neuroprotective mechanism is associated with regulating lipid peroxidation, antioxidant activity, essential elements, Zn/Cu ratio, and hazardous metal Pb.

## 1. Introduction

Epigallocatechin gallate (EGCG) is a natural compound that mainly exists in fruits and plants to protect against environmental stress [1]. The rat model displays that EGCG possesses the beneficial properties of anti-inflammation and anti-oxidation [1,2,3]. Therefore, EGCG has been shown to exert neuroprotective efficacies that result in increased numbers of neurons and the recovery of motor functions in spinal cord injury and sciatic nerve crush injuries [4,5,6].

Cerebral ischemia results from the occlusion of cerebral arteries, blocking blood flow to the affected brain tissues. Cerebral ischemia-generated reactive oxygen species (ROS) can result in elevated oxidative stress and further brain injury. Recent studies have revealed that the biochemical changes resulting in brain cell death after cerebral ischemia occur in an orderly series of steps: ionic perturbation, oxidative stress, and inflammation [1,2,7,8]. Thus, ionic perturbation plays a crucial role in the early stage of initiating brain cell death after cerebral ischemia. Cerebral ischemia not only generates substantial ROS (superoxide radicals, hydrogen peroxide, and hydroxyl radicals), but also promotes deleterious lipid peroxidation [7,8]. Lipid peroxidation is mediated by ROS attack of polyunsaturated fatty acids (PUFA), further increasing oxidative damage and even brain cell death [8]. The brain characteristics of high aerobic metabolism, high PUFA component, and less antioxidant capacity make it highly vulnerable to ROS attack [7]. As already mentioned, the beneficial properties of EGCG in anti-oxidation and anti-inflammation make it a promising agent for the promotion of neurobehavioral recovery after injury [1,2,3,5,6].

Superoxide dismutase (SOD) and catalase (CAT) are important antioxidants that are pivotal for protecting the brain from oxidative attack [7,8]. Detoxification by SOD occurs through its catalysis of reactions that convert toxic superoxide radicals into hydrogen peroxide, and CAT then converts hydrogen peroxide into water [7]. Thus, an inverse association has been documented between decreased SOD and CAT activity and elevated oxidative stress in the brain [8].

Maintaining appropriate brain concentrations of several essential elements is required for normal brain functions [7]. Significant attention has been focused on magnesium (Mg), zinc (Zn), and selenium (Se) because of their innate anti-oxidant and anti-inflammatory properties [7,8]. In addition, iron (Fe) and copper (Cu) are essential for brain functions but excess concentrations can generate deleterious ROS via the Fenton reaction [7,8]. On the other hand, the hazardous metal lead (Pb) is not only ubiquitous, but also presents as a contaminant in the environment [8,9,10]. Due to its wide applications in industry and human use, exposure to Pb is virtually inescapable [8,9,10]. Pb not is not only involved in any biological functions in animals and humans, but also accumulates in the body, primarily in the brain. It is evidenced that Pb-induced neurotoxicity is associated with elevating oxidative stress caused by ROS generation and antioxidant enzyme depletion [8,9,10].

Despite numerous studies exploring the mechanism underlying EGCG neuroprotection, whether this mechanism involves the regulation of brain concentrations of essential elements and hazardous metal is still unknown and is the aim of this present study.

## 2. Materials and Methods

### 2.1. Animal Treatment and Cerebral Ischemic Surgery

In total, forty male Sprague-Dawley rats weighing from 250 to 300 g were enrolled in this present work. The experimental animals were purchased from BioLASCO, Taipei, Taiwan. In order to stabilize the physiological conditions of animals, rats were housed in stainless-steel mesh cages under controlled conditions. The relative humidity was controlled in the range of 50 ± 20% and the temperature was controlled at 22 ± 2 °C and maintained at 12-h light-dark cycle for 7 days. Rats were randomly assigned into four groups of 10 each as below: control (rats were intraperitoneally treated with normal saline for consecutive 10 days), ligation (rats were given with normal saline for consecutive 10 days followed by ligation of the right common carotid artery (CCA) and the right middle cerebral artery (MCA) for 60 min on day 10th), EGCG (rats were intraperitoneally given with EGCG (Sigma-Aldrich, Merck, Germany) at a dosage of 50 mg/Kg once daily for consecutive 10 days), and prevention (rats were intraperitoneally injected with 50 mg/Kg of EGCG once daily for 10 days followed by ligation of the CCA and the MCA for 60 min). The cerebral ischemic operation was performed at the rat age of eight-week that the right CCA was exposed and carefully isolated from the vago-sympathetic trunks followed by loosely encircled for further ligation. After performing a midline incision, the skull bone was craniectomized to expose the right MCA. Meanwhile, an 8–0 suture (blue monofilament polypropylene, DG, Davis-GECK, Wayne, NJ, USA) was used to position so as to encircle the MCA for further ligation. For CCA ligation, a midline neck incision was made and the right CCA was exposed and carefully separated from the vago-sympathetic trunks. The right CCA was loosely encircled with a 4-O suture for later occlusion. At the end of ligation surgery, the right brain cortex tissues were immediately collected and homogenized, followed by harvesting of the supernatants for further biochemical analysis. Experimentally, all performed protocols throughout this animal experiment were approved in advance by the Institutional Animal Care and Use Committee (107-CTUST-013, IACUC) of Central Taiwan University of Science and Technology in Taiwan.

### 2.2. Malondialdehyde (MDA) Analysis in the Homogenates of Brain Cortex

The malondialdehyde (MDA) level was measured to evaluate ROS-mediated lipid peroxidation in the brain cortex. In this work, 0.2 g of the obtained right brain cortex tissues were homogenized with the volume of 5 mL of ice KCl (154 mM) solution via Teflon pestles homogenizers followed by centrifuge at 4 °C for 10 min at the speed of 650 g, and the supernatant was immediately harvested. For the MDA analysis, 200 μL of the supernatant was mixed with 3 mL of the H_3_PO_4_ and 800 μL of the KCl solution and vortexed carefully. In addition, the standard solution of 1,1,3,3-tetra ethoxy propane was applied to react with the thiobarbituric acid (TBA) substance followed by boiling for 60 min. Experimentally, 4 mL of the butanol was carefully added into the solution and mixed for 5 min followed by harvesting the supernatant for MDA analysis. Basically, the detective principle is to measure the intensity of the generated pink color via the reaction of MDA with TBA substance. The MDA level in the right brain cortex homogenates was measured by spectrophotometry at the wavelength of 532 nm (U-1900, Hitachi, Japan).

### 2.3. Measurement of SOD and CAT Activity in the Brain Cortex Homogenates

In this study, a total of 0.2 g of the right brain cortex tissue was harvested for measuring the antioxidant activity of SOD and CAT. Briefly, the SOD activity was analyzed according to the detective procedures for the Cayman’s superoxide dismutase assay kit purchased from Cayman Chemical Company, Ann Arbor, MI, USA. The analytical principle is that the xanthine oxidase reacts with the hypoxanthine so as to produce the superoxide radical (O_2_^•−^). The generated superoxide radical interacts with the tetrazolium salt, and the antioxidant activity of SOD was analyzed by spectrophotometry (Thermo Scientific Multiskan Spectrum, Ann Arbor, MI, USA). The enzyme activity of SOD was expressed in terms of U per mg of protein concentration. For the analysis of CAT activity, the commercial kit was purchased from Cayman Chemical Company (Ann Arbor, MI, USA). For the analytical procedure, the methanol was reacted with hydrogen peroxide under the catalyzation of the CAT enzyme to produce the product of formaldehyde. The generated formaldehyde interacted with the chromogen of 4-amino-3-hydrazino-5-mercapto-1,2,4-triazole formaldehyde, and the CAT activity was detected by spectrophotometry (Thermo Scientific Multiskan Spectrum, Ann Arbor, MI, USA). The enzyme activity was expressed in terms of U per mg of protein concentration.

### 2.4. Determination of Essential Elements and Hazardous Metal in Brain Cortex Homogenates

For analyzing essential elements and hazardous metal, a total of 0.2 g of the right brain cortex sample was used and wet digestion was performed overnight by adding 4 mL of ultra-pure grade nitric acid. After complete digestion, the homogenous suspension was used to detect the concentration of Mg, Zn, Se, Fe, Cu, and Pb. To avoid any metal contamination throughout the analytical procedures, all experimentally used containers were soaked with 50% nitric acid, rinsed with water, followed by drying in an oven at the temperature of 50 °C, ready for experimental use. Additionally, in order to enhance the analytical sensitivity for the determination of Se element, a specific hollow cathode, so-called super-lamp (Victoria, Braeside, Australia) was used in this study. The standard solution of each element and hazardous metal was dissolved in the concentration of 0.1 mol/L nitric acid solution purchased from Merck, Darmstadt, Germany. The SavantAA Z graphite furnace atomic absorption spectrophotometer with longitudinal Zeeman effect background correction and PAL4000 auto-sampler system was used in this study (GBC Scientific Equipment Pty Ltd., Darmstadt, Australia).

### 2.5. Measurement of Protein Concentration in the Brain Cortex Homogenates

In this present study, the commercial BioChain protein assay kit, purchased from San Francisco, CA, USA, was used to analyze the protein concentration. The analytical method of the protein concentration assay kit was improved by the Coomassie Blue G method. In brief, the analytical principle of this method is that the reagent was reacted with the protein to produce a blue color complex. The color intensity of the generated blue color complex is paralleled with the protein concentration. Finally, the protein level was assayed by spectrophotometry (Thermo Scientific Multiskan Spectrum, USA) at the wavelength of 595 nm experimentally.

### 2.6. Statistical Analysis

All the obtained values from the experimental analysis were expressed as mean ± S.D. The obtained data were analyzed by the statistical method of Kruskal–Wallis one-way analysis of variance (ANOVA). Basically, if the experimental values exhibit significant differences among groups, Fisher’s least significant difference (FLSD) was applied to compare each group. If the *p*-value was less than 0.05, the statistical differences were considered significant in this present study. a: *p* < 0.05, vs. control; b: *p* < 0.05, vs. ligation.

## 3. Results

### 3.1. Malondialdehyde (MDA) Concentration in the Brain Cortex Homogenates

**Figure 1:**
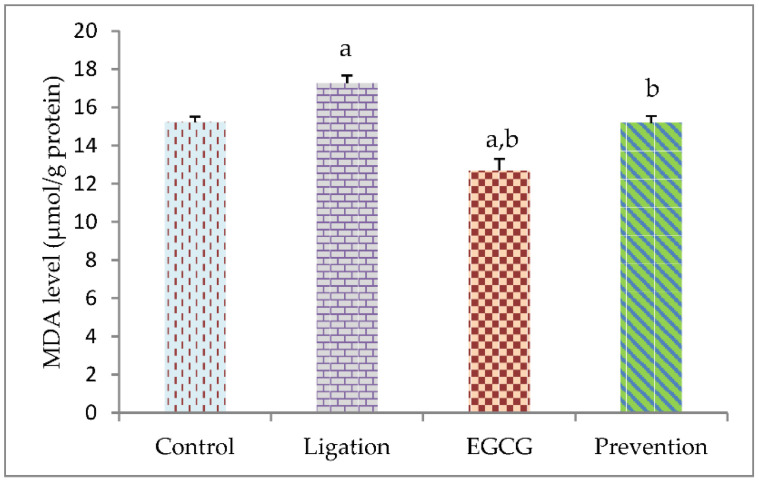
Malondialdehyde (MDA) level in the brain cortex homogenates. The experimental data were expressed as mean ± S.D. The statistical method of Kruskal–Wallis one-way analysis of variance (ANOVA) followed by the Fisher’s least significant difference test was used in this experiment. Difference of statistic was considered significant at *p* < 0.05. a: *p* < 0.05 vs. control subject; b: *p* < 0.05 vs. ligation subject.

Figure 1 illustrates the experimental results regarding the MDA level in the brain cortex homogenates correlated to the status of oxidative injury. The MDA level was significantly increased (*p* < 0.05) in the ligation group as compared to the control group. Conversely, pretreatment of rats with EGCG before cerebral ischemic insult obviously (*p* < 0.05) attenuated the MDA concentration in the prevention subject as compared to the ligation group.

### 3.2. Antioxidant Enzyme Activities of SOD and CAT in the Brain Cortex Homogenates

**Figure 2:**
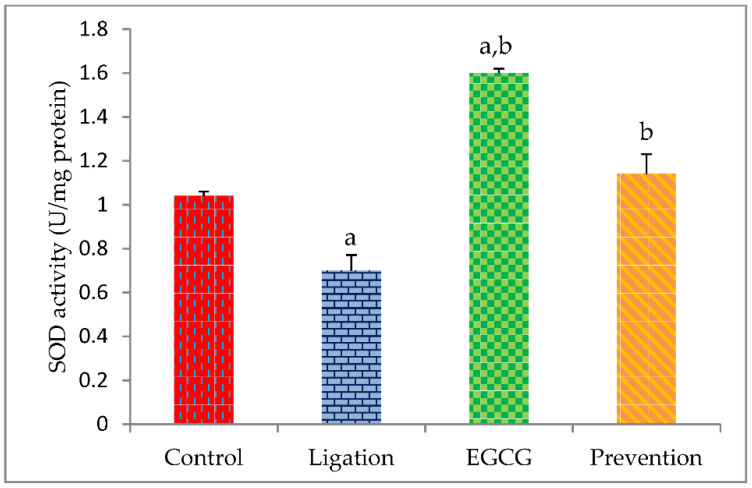
Antioxidant activity of SOD in the brain cortex homogenates. The experimental values were expressed as mean ± S.D. The statistical method of Kruskal–Wallis one-way analysis of variance (ANOVA) followed by the Fisher’s least significant difference test was used in this experiment. Difference of statistic was considered significant at *p* < 0.05. a: *p* < 0.05 vs. control subject; b: *p* < 0.05 vs. ligation subject.

In comparison with the control group, the SOD activity was significantly decreased in the ligation group (Figure 2). Pretreating rats with EGCG before ligation obviously raised the SOD activity in the prevention subject as compared to the ligation group.

**Figure 3:**
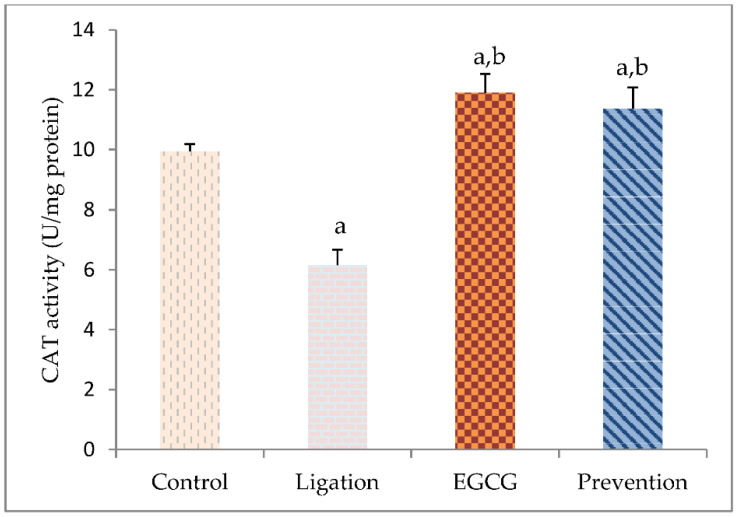
Enzyme activity of CAT in the brain cortex homogenates. The obtained data were expressed as mean ± S.D. The statistical method of Kruskal–Wallis one-way analysis of variance (ANOVA) followed by the Fisher’s least significant difference test was used in this study. Difference of statistic was considered significant at *p* < 0.05. a: *p* < 0.05 vs. control subject; b: *p* < 0.05 vs. ligation subject.

Experimentally, the CAT value was significantly diminished in the prevention subject as compared to the ligation group (Figure 3). However, pretreating rats with EGCG before artery ligation markedly raised the CAT activity in the prevention subject as compared to the ligation group.

### 3.3. Essential Elements and Zinc-Copper Ratio (Zn/Cu Ratio) in the Brain Cortex Homogenates

**Figure 4:**
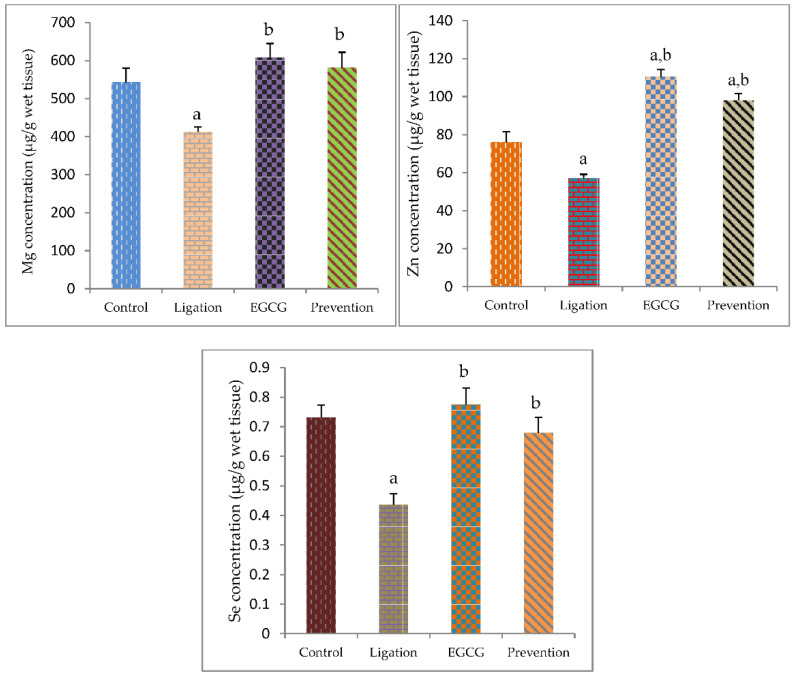
Concentration of essential element Mg, Zn, and Se in the brain cortex homogenates. The experimental values were expressed as mean ± S.D. The statistical method of Kruskal–Wallis one-way analysis of variance (ANOVA) followed by the Fisher’s least significant difference test was used in this present study. Difference of statistic was considered significant at *p* < 0.05. a: *p* < 0.05 vs. control subject; b: *p* < 0.05 vs. ligation subject.

In the ligation group compared to the control group, a significant decrease of the brain cortex Mg level was found (Figure 4). Pretreatment of rats with EGCG prior to cerebral ischemia significantly elevated the Mg values as compared to the ligation group.

The value of essential element Zn was significantly diminished in the ligation subject as compared to the control group (Figure 4). Interestingly, pretreating rats with EGCG prior to artery ligation prominently increased the Zn level in the prevention subject as compared to the ligation group. The Se level in the right brain cortex was markedly declined in the ligation group as compared to the control group (Figure 4). Pretreating rats with EGCG before ischemic insult obviously enhanced the Se level in the prevention group as compared to the ligation group.

**Figure 5:**
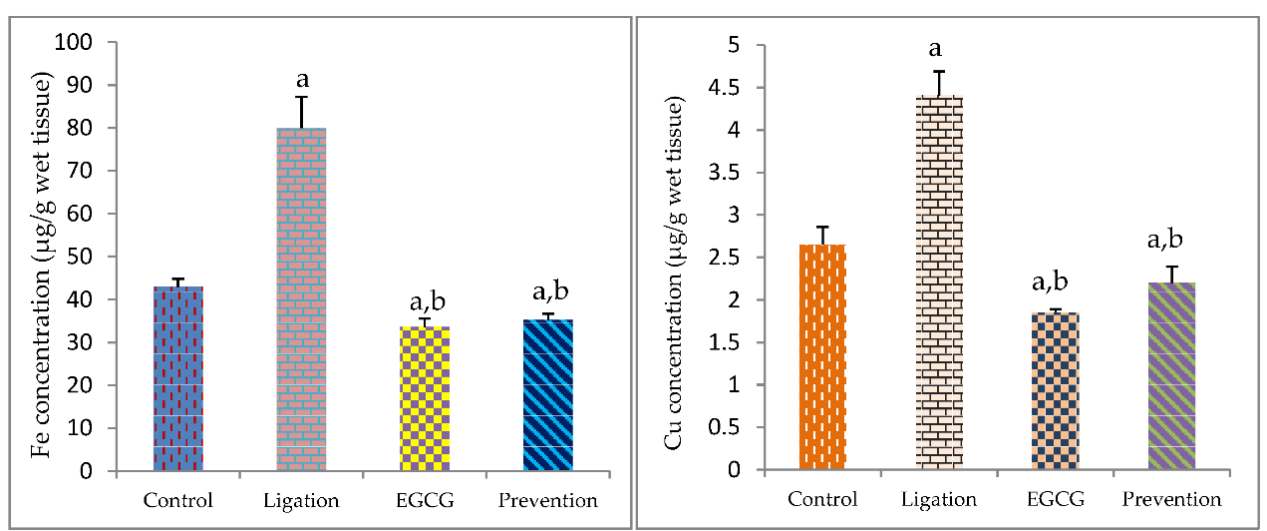
The concentration of essential element Fe and Cu in the brain cortex homogenates. The experimental values were expressed as mean ± S.D. The statistical method of Kruskal–Wallis one-way analysis of variance (ANOVA) followed by the Fisher’s least significant difference test was used in this present study. Difference of statistic was considered significant at *p* < 0.05. a: *p* < 0.05 vs. control subject; b: *p* < 0.05 vs. ligation subject.

The Fe level in the ligation group was markedly higher as compared to the control group (Figure 5). Pretreatment of animals with EGCG prior to ischemia significantly decreased the Fe level in the prevention group as compared to the ligation group. Figure 5 indicates the obtained results regarding the Cu level in the brain cortex homogenates. The Cu level in the ligation group was statistically higher as compared to the control group. However, pretreating rats with EGCG before ischemic injury obviously reduced the Cu level in the prevention group as compared to the artery ligation group.

**Figure 6:**
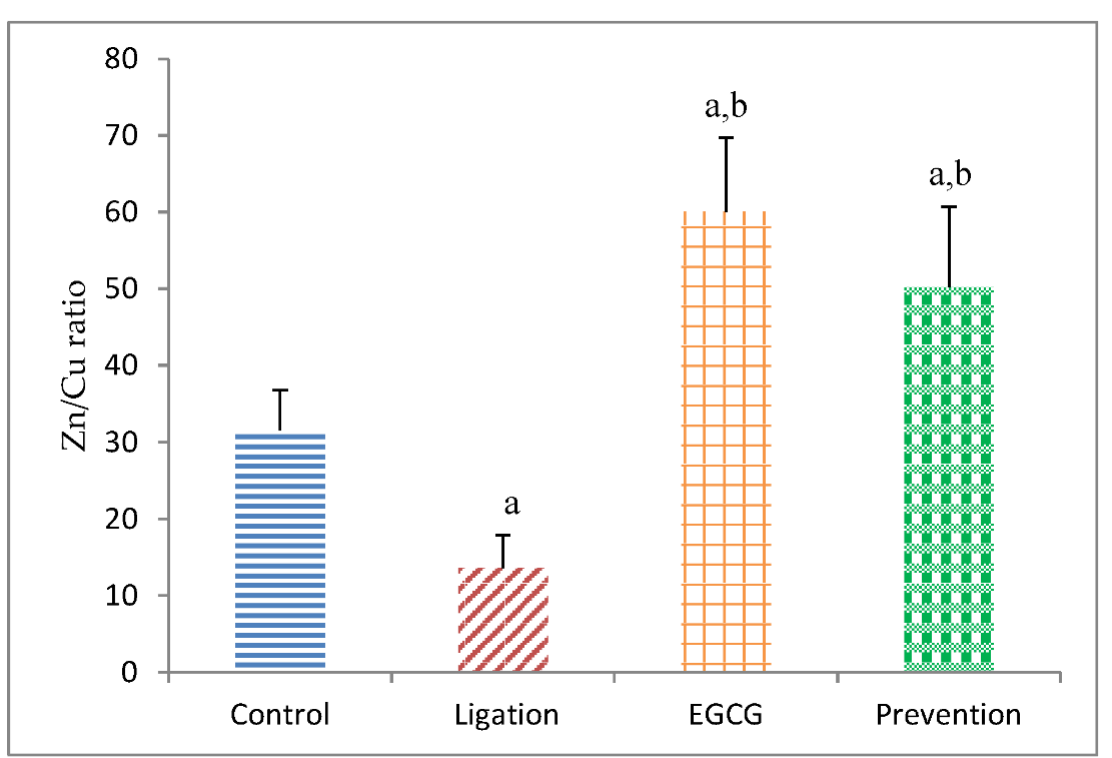
The Zn/Cu ratio in the brain cortex homogenates. The obtained values were expressed as mean ± S.D. The statistical method of Kruskal–Wallis one-way analysis of variance (ANOVA) followed by the Fisher’s least significant difference test was applied in this present study. Difference of statistic was considered significant at *p* < 0.05. a: *p* < 0.05 vs. control subject; b: *p* < 0.05 vs. ligation subject.

Figure 6 shows the experimental data regarding the Zn/Cu ratio in the right brain cortex homogenates correlated to the status of oxidative stress and nutrition. The Zn/Cu ratio in the ligation group was markedly lower as compared to the control group. Interestingly, pretreatment of animals with EGCG before ischemic insult significantly increased the Zn/Cu ratio in the prevention subject as compared to the ligation group.

**Figure 7:**
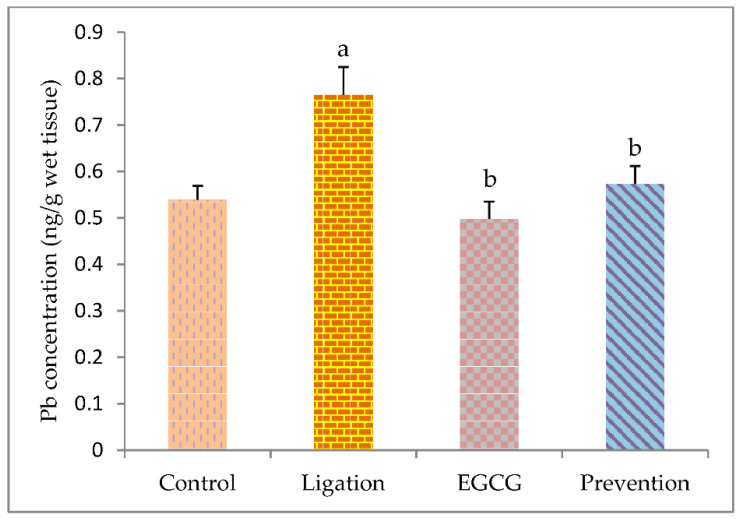
The concentration of hazardous metal Pb in the brain cortex homogenates. The experimental data were expressed as mean ± S.D. The statistical method of Kruskal–Wallis one-way analysis of variance (ANOVA) followed by the Fisher’s least significant difference test was used in this study. Difference of statistic was considered significant at *p* < 0.05. a: *p* < 0.05 vs. control subject; b: *p* < 0.05 vs. ligation subject.

Figure 7 illustrates the obtained values concerning the concentration of hazardous metal Pb in the brain cortex homogenates. The Pb level was significantly increased (*p* < 0.05) in the artery ligation group as compared to the control group. Specifically, pretreating rats with EGCG before cerebral ischemic injury obviously (*p* < 0.05) attenuated the Pb concentration in the prevention group as compared to the ligation subject.

## 4. Discussion

Our present findings highlight that cerebral ischemia not only results in brain tissue decreases in Mg, Zn, Se, and the Zn/Cu ratio and antioxidant activity of SOD and CAT, but also increases concentrations of MDA, Fe, Cu, and Pb. Notably, pretreating rats with EGCG before ischemic insult significantly reversed these effects. EGCG is known for its wide array of beneficial properties to humans and animals in terms of anti-oxidation and anti-inflammation [1]. Thus, multiple lines of evidence from animal models suggest that EGCG exhibits a wide range of neuroprotection to improve various neurological disorders, such as ischemic stroke, Huntington’s, and Alzheimer’s diseases [1,11,12]. Meanwhile, research reveals that EGCG is capable of improving ROS-mediated oxidative stress and inflammatory responses so as to effectively reduce further oxidative injury in rats with spinal cord injury [1,2,3]. Another animal study indicates neurological diseases of contusive spinal cord injury and neuropathic pain can be mitigated by EGCG therapy [11]. Furthermore, EGCG exerts anti-inflammatory ability to effectively alleviate spinal cord trauma in rat models [12]. The animal experiment proposes that along with its anti-inflammatory property, EGCG significantly promotes neuronal generation after spinal cord injury via reducing the inflammatory cytokines IL-2, IL-6, IL-1β, and TNF-α so as to significantly attenuate the situation of spinal cord injury [13]. On the other hand, sciatic nerve crush injury-induced neurobehavioral and morphological disorders can be alleviated by EGCG via enhancing total antioxidant capacity [14]. Additionally, in vivo studies demonstrate that supplementation of EGCG to streptozotocin-nicotinamide-induced diabetic rats with cardiomyopathy not only markedly mitigates ROS-mediated lipid peroxidation, inflammatory cytokines, fibrosis, and cell death, but also significantly enhances the antioxidant activity of SOD and CAT [15,16]. As mentioned above, it is notable that neuroprotection of EGCG is correlated with the properties of anti-oxidation, anti-inflammation, and antioxidant enhancement. Our present finding reveals that pretreating rats with EGCG before cerebral ischemic injury significantly attenuates ROS-mediated lipid peroxidation in the prevention group and the result of this present study is in agreement with the preceding investigation. 

Cerebral ischemia is characterized by the occlusion of cerebral artery caused by thrombi or embolism to interrupt blood flow into the brain [17,18]. Ischemic stroke is a pathological situation attended with elevated oxidative stress resulting from ROS generation [17,18]. The previous study indicates that the generated ROS results from ischemic stroke significantly attenuate antioxidant capacity in the affected brain [17,18]. In fact, the poly-unsaturated fatty acid (PUFA) is the major component of the cell membrane and is the primary target of ROS attack because of its double bond structure to transform chemical and geometric structures of the cell membrane [19,20]. Consequently, membrane pore formation and destroyed barrier function lead to cell death. It has been suggested that the brain is highly vulnerable to ROS attack because of the characters of PUFA content, high aerobic metabolism, and less antioxidant capacity [7,8,17,18]. Our present findings reveal that pretreating rats with EGCG before an ischemic event significantly enhances the antioxidant activity of SOD and CAT. Obviously, our finding is in line with the preceding study, suggesting that the beneficial effect of EGCG on the ischemic brain cortex during cerebral ischemia is associated with directly elevating antioxidant enzyme activity.

Despite the numerous and continuous achievements over the years in exploring the mechanisms of EGCG underlying neuroprotection, it has not yet been elucidated so far whether the neuroprotective mechanism of EGCG is associated with the modulations of essential elements and hazardous metal during cerebral ischemic injury. A recent study reveals that cerebral ischemia-induced brain cell death is implicated with three dominant mechanisms and suggests that ionic perturbation is the first, followed by oxidative stress, and inflammation is the last one [21]. In fact, the occurrence of ionic perturbation is developed within a few minutes after ischemic insult [21]. It means that ionic perturbation plays the first and crucial role in initiating brain cell death during the ischemic phase. Therefore, understanding the situation of ionic perturbation during cerebral ischemia is helpful to elucidate the relationship between the alterations of essential elements, hazardous metal, and cerebral ischemic injury. 

Mg is known as the most abundant intracellular cation and is involved in a wide range of biological functions in all living organisms [18]. Mg acts as a cofactor for more than 300 enzymes and involves multiple cellular functions, such as modulating energy metabolism, regulating ATP synthesis, reducing neuronal excite-toxicity, acting as a calcium blocker, protecting cells from oxidative attack, and ameliorating inflammatory response [19]. Another beneficial effect of Mg is to specifically block the n-methyl-D-aspartate (NMDA) receptor [17,19]. Thus, Mg deficiency may lead to NMDA over-activation, resulting in further neurotoxicity [19]. In this present study, the cerebral ischemic injury resulted in decreased Mg but increased lipid peroxidation in the affected brain. Interestingly, pretreating rats with EGCG before ischemia significantly reversed these detrimental effects. Notably, the beneficial effect of EGCG is to effectively increase the Mg levels. Mg exerts its natural antioxidant properties to attenuate ROS-mediated lipid peroxidation in the ischemic brain cortex. Our finding is consistent with that of our preceding study in which pretreating gerbils with magnesium sulfate before ischemic stroke significantly attenuated excitotoxic glutamate levels so as to decrease neurotoxicity and brain infarct volumes [20]. Additionally, Mg can decrease calcium influx via its calcium blocker property, thereby ameliorating calcium-mediated cell damage [19]. In vivo studies reveal an inverse association between Mg level and lipid peroxidation status during focal cerebral ischemia [17,18]. Further, clinical research suggests that patients with lower serum Mg concentrations during the ischemic stroke are vulnerable to neurologic deterioration and worse outcomes [22]. Altogether, our finding suggests that the neuroprotective mechanism of EGCG during cerebral ischemia is to increase the Mg level so as to attenuate ROS-mediated lipid peroxidation in the ischemic brain. 

Essential element Zn is important in numerous physiological functions in humans, including anti-oxidation, anti-inflammation, brain development, and wound healing [8,19]. Our preceding study reveals that a positive correlation was found between the Zn level and the antioxidant activities of SOD and CAT in the ischemic brain cortex during cerebral ischemia [7]. Animal studies suggest that pretreatment of rats with resveratrol before ischemic stroke significantly enhances Zn level so as to increase the antioxidant enzyme activity of SOD and CAT in the ischemic brain [7,8]. An animal study shows that Zn supplementation not only elevates SOD and CAT activity in the serum [7], but also attenuates inflammatory factors during heat stress [23]. An in vivo experiment indicates the beneficial effect of Zn in human renal tubule cells whereby a reduced Zn level can down-regulate the expression of the protein of nuclear factor erythroid 2–related factor 2 (Nrf2) and greatly decrease the antioxidant activity of SOD and glutathione S-transferase [24]. In this present study, cerebral ischemia results in a marked decrease in Zn level and increased lipid peroxidation. However, pretreating rats with EGCG before ischemia significantly reversed these results. Our finding confirms that the neuroprotective mechanism of EGCG involves the elevation of Zn levels. Under this situation, Zn exerts its natural anti-oxidant and anti-inflammatory actions to effectively attenuate ROS-mediated lipid peroxidation in the ischemic brain cortex and this present finding is in line with the previous investigation. 

It has been evidenced that essential trace element Se exerts multiple beneficial effects, including ROS scavenging, anti-inflammation, anti-oxidation, and increasing the antioxidant capacity [25]. Conversely, lower Se levels weaken the antioxidant capacity, leading to elevated oxidative stress and further cellular injury [25]. An in vivo study reports that cisplatin-induced oxidative renal injury was ameliorated by Se supplementation through directly reducing ROS-mediated lipid peroxidation and increasing the antioxidant activity of SOD and CAT [26]. A clinical study reveals a negative correlation between serum Se level and the occurrence of acute ischemic stroke [27]. Experimental investigation with rats demonstrates that Se supplementation displays protective effects against patulin-conducted brain lesion in mice via increases in GSH-related enzyme activity [28]. Further, pretreating Alzheimer’s disease model rats with sodium selenate markedly decreased neurodegeneration and neurological deficits [29]. Finally, supplementation of Se to lymphocytes obtained from Alzheimer’s disease patients effectively decreases ROS generation and increases antioxidant activity [30]. In this current study, pretreating rats with EGCG before ischemic stroke significantly increased the Se concentration in the ischemic brain. The trend of our experimental finding is consistent with previous reports suggesting that the mechanism underlying the neuroprotection of EGCG involves increasing the Se concentration. As a result, Se can exert its innate property of anti-inflammation and anti-oxidation to mitigate ROS-mediated lipid peroxidation during ischemic stroke.

Recent studies have revealed that phenolic hydroxyl groups of EGCG exert a metal chelating effect [31,32,33]. A previous study proposes that ischemic stroke results in Fe overload in the affected brain cortex [8]. Additionally, ischemia-generated hydrogen peroxide can spontaneously react with Fe to generate toxic hydroxyl radicals through the Fenton reaction, causing further oxidative brain injury and even cell death [34,35]. EGCG has gained attention because of its metal chelating property and has been used to treat metal-related neurological diseases [36]. We observed here that pretreating rats with EGCG before ischemic stroke markedly decreases the Fe level in the ischemic brain cortex. Our result is consistent with the previous reports and suggests that the EGCG effectively chelates the Fe element so as to significantly attenuate Fe-induced Fenton reaction and further detrimental lipid peroxidation.

Cu is essential for the brain and is mainly involved in the biological functions in the synthesis of neuropeptides, catecholamine, and cytochrome oxidase [37,38]. Similar to the Fe, Cu overload is toxic to cells because it initiates the deleterious Fenton reaction to generate toxic hydroxyl radicals, resulting in further oxidative cellular injury and cell death [18,37]. A clinical study reveals a relationship between elevated plasma Cu levels and the etiology of Alzheimer’s disease [38]. A variety of animal studies reveal the fact that cerebral ischemic injury results in an obviously elevated Cu concentration in the affected brain cortex tissues [8,17,18,39]. Likewise, our present finding proposes that cerebral ischemic insult leads to significant Cu overload in the ischemic brain. Importantly, we observed that pretreating rats with EGCG before ischemia markedly declined the Cu element. We suggest that this beneficial effect of EGCG likely results from its chelating property. Thereby, it is notable to state this beneficial effect whereby maintaining relatively lower Cu levels during cerebral ischemia effectively decreases the Cu-induced Fenton reaction as well as ROS-mediated detrimental lipid peroxidation effect.

A recent study revealed that the Zn/Cu ratio is a useful biomarker for assessing oxidative stress, nutritional status, and inflammation [40]. A study of ischemic stroke pathophysiology shows a marked correlation between lower Zn/Cu ratio and larger brain infarct size in stroke patients [40]. Furthermore, clinical study suggests that patients with acute ischemic stroke not only possess poor nutritional status, but also show a decreased blood Zn/Cu ratio [40]. Other clinical evidence demonstrates a significant association between lower Zn/Cu ratio and elevated concentration of the inflammatory cytokine of C-reactive protein (CRP) in men with cerebral ischemic lesions [41]. In this present study, cerebral ischemic injury results in a lower Zn/Cu ratio but pretreating rats with EGCG before ischemia obviously reversed this detrimental phenomenon. Clearly, the neuroprotective effect of EGCG on the ischemic brain cortex is related to decreased Cu and increased Zn concentrations, resulting in a higher Zn/Cu ratio as a consequence. Based on our experimental findings, we suggest that a higher Zn/Cu ratio is correlated to attenuated oxidative stress and oxidative brain injury. As a result, lipid peroxidation decreased and antioxidant capacity increased.

Accumulating evidence indicates that the mechanism underlying Pb toxicity involves elevated oxidative stress due to ROS production and antioxidant capacity depletion [42,43]. In this present experiment, we observed that the brains of rats pretreated with EGCG before ischemic injury had lower Pb concentrations than did those from untreated ischemic injury rats. Previous studies demonstrate the biological toxicity of Pb as exposure of the rat brain to hazardous metal Pb is associated with decreasing the antioxidant activity of SOD and CAT and increasing ROS-mediated lipid peroxidation [44,45]. Furthermore, a significant change in biogenic amine levels was observed in the brains of lead-poisoned rats [46]. Altogether, it is clear that Pb-induced neurotoxicity is correlated with ROS generation combined with antioxidant capacity depletion. As already mentioned, one of the protective mechanisms of oxidative injury in the brain by EGCG is attributed to its metal chelating property [36,47]. Similar to the active polyphenol constituent of EGCG, pretreating rats with the polyphenol compound resveratrol before cerebral ischemic insult significantly decreases Pb levels in the ischemic brain, and this effect correlates with its chelating property [8]. Our present finding is in accordance with these preceding findings, suggesting that pretreatment of rats with EGCG effectively chelates hazardous metal Pb, attenuating Pb-induced ROS generation, decreasing ROS-induced lipid peroxidation and further neurotoxicity. Specifically, this present study is the first to report that the mechanism underlying neuroprotection by EGCG involves decreasing the hazardous metal Pb levels in the ischemic brain cortex.

## 5. Conclusions

EGCG confers neuroprotection through multiple mechanisms. This present study is the first to highlight that EGCG modulates the levels of essential elements and hazardous metals in the brain cortex during cerebral ischemic injury. Altogether, our findings demonstrate that the neuroprotective effect of EGCG involves the attenuation of ROS-mediated lipid peroxidation and the Fenton reaction-associated elements Fe and Cu, increasing the antioxidant activity of SOD and CAT, the concentrations of the natural antioxidant elements Mg, Zn, and Se, and the Zn/Cu ratio. More critically, EGCG exerts its chelating property to significantly chelate Fe, Cu, and Pb, thereby obviously ameliorating Fe-, Cu-, and Pb-induced ROS generation, lipid peroxidation, and further oxidative brain injury. The limitation of this present study is the lack of cerebral blood flow measurement to precisely confirm the status of ischemic surgery. In addition, the detailed mechanism by which EGCG increases antioxidant-related elements of Mg, Zn, and Se will require further elucidation.

## Data Availability

The data presented in this study are available in this manuscript.
